# Siponimod (BAF312) Activates Nrf2 While Hampering NFκB in Human Astrocytes, and Protects From Astrocyte-Induced Neurodegeneration

**DOI:** 10.3389/fimmu.2020.00635

**Published:** 2020-04-08

**Authors:** Emanuela Colombo, Claudia Bassani, Anthea De Angelis, Francesca Ruffini, Linda Ottoboni, Giancarlo Comi, Gianvito Martino, Cinthia Farina

**Affiliations:** Institute of Experimental Neurology (INSpe), Division of Neuroscience, San Raffaele Scientific Institute, Milan, Italy

**Keywords:** astrocytes, BAF312, fingolimod, neurodegeneration, neuroinflammation, NFκB, Nrf2, siponimod

## Abstract

Multiple sclerosis (MS) is an inflammatory neurodegenerative disease of the central nervous system (CNS) with heterogeneous pathophysiology. In its progressive course oligodendrocyte and neuroaxonal damage is sustained by compartmentalized inflammation due to glial dysregulation. Siponimod (BAF312), a modulator of two sphingosine-1-phosphate (S1P) receptors (S1P1 and S1P5) is the first oral treatment specifically approved for active secondary progressive MS. To address potential direct effects of BAF312 on glial function and glia-neuron interaction, we set up a series of *in vitro* functional assays with astrocytes generated from human fibroblasts. These cells displayed the typical morphology and markers of astroglia, and were susceptible to the action of inflammatory mediators and BAF312, because expressing receptors for IL1, IL17, and S1P (namely S1P1 and S1P3). Targeting of S1P signaling by BAF312 inhibited NFκB translocation evoked by inflammatory cytokines, indicating a direct anti-inflammatory activity of the drug on the human astrocyte. Further, while glia cells exposed to IL1 or IL17 downregulated protein expression of glutamate transporters, BAF312-treated astrocytes maintained high levels of GLAST and GLT1 regardless of the presence of inflammatory mediators. Interestingly, despite potential glial susceptibility to S1P signaling via S1P3, which is not targeted by BAF312, NFκB translocation and downregulation of glutamate transporters in response to S1P were inhibited at similar levels by BAF312 and FTY720, another S1P signaling modulator targeting also S1P3. Accordingly, specific inhibition of S1P1 via NIBR-0213 blocked S1P-evoked NFκB translocation, demonstrating that modulation of S1P1 is sufficient to dampen signaling via other S1P receptors. Considering that NFκB-dependent responses are regulated by Nrf2, we measured activation of this critical transcription factor for anti-oxidant reactions, and observed that BAF312 rapidly induced nuclear translocation of Nrf2, but this effect was attenuated in the presence of an inflammatory milieu. Finally, *in vitro* experiments with spinal neurons exposed to astrocyte-conditioned media showed that modulation of S1P or cytokine signaling in astrocytes via BAF312 prevented neurons from astrocyte-induced degeneration. Overall, these experiments on human astrocytes suggest that during neuroinflammation targeting of S1P1 via BAF312 may modulate key astrocyte functions and thereby attain neuroprotection indirectly.

## Introduction

Multiple sclerosis (MS) is a complex, highly debilitating inflammatory disease of the central nervous system (CNS) and represents the most common cause of neurological disability in young adults (1). Most of the available drugs display efficacy in the relapsing-remitting (RR-MS) form of the disease (2), where frequent waves of infiltrating immune cells into the CNS lead to demyelination, but not in progressive MS, where oligodendrocyte and neuroaxonal damage is sustained by compartmentalized inflammation due to glial dysregulation (3). After several years of disease most of the RR-MS patients enter the progressive stage (called secondary progressive MS, SP-MS), characterized by steady accumulation of disability in absence of acute clinical events (4). Sphingosine 1-phosphate (S1P)–S1P receptor axis is a known pharmacological target in MS, due to its key role in the regulation of immune cell migration from peripheral lymphoid organs to CNS (5). S1P signals through five G protein-coupled receptors (S1P1-5), which are widely expressed and control several cellular processes, such as growth, survival and differentiation (5). Neurons and glia cells in the CNS may bear S1P receptors (6, 7), opening to the possibility of interfering with events occurring in the nervous tissue via targeting S1P signaling pathway. Fingolimod (FTY720), the first oral therapy approved for RR-MS, is a prodrug that, after activation by phosphorylation, binds to all S1P receptors with the exception of S1P2 (8), and thereby induces lymphopenia (9), reduces the inflammatory activation of circulating and CNS-resident myeloid cells (10–13), and blocks astrocyte activation during neuroinflammation (7, 14, 15). Despite the lack of efficacy for fingolimod in progressive MS (16), the potential neuroprotective effects due to the blockade of S1P-S1P receptor axis in CNS prompted the development of novel S1P receptor modulators which work as active drugs. The recent phase-3 EXPAND trial demonstrated that oral administration of siponimod (BAF312), which targets S1P1 and S1P5 (17), attenuates the risk of disability progression in SP-MS, with a major effect in those patients with inflammatory disease (18). For this reason the European Medicines Agency recommended BAF312 as first oral treatment for active SP-MS in November 2019^1^. BAF312 treatment significantly hinders lesion enlargement and brain atrophy after 12 months (18), demonstrating relevant protective properties in CNS tissue via mechanisms which remain to be clarified. *In vitro* models for human astrocytes can be generated from readily accessible cells, such as fibroblasts, and provide the unprecedented possibility to explore the contribution of this glia cell population to human diseases, study its interaction with neuronal cells and test potential neuroprotective drugs. To address direct effects of BAF312 on glial function and glia-neuron interaction, we generated human astrocytes from reprogrammed fibroblasts and set up a series of *in vitro* assays to verify whether BAF312 may hamper glial inflammatory activity and support physiological and anti-oxidant functions of the astrocyte.

## Materials and Methods

### Fibroblast Reprogramming and Differentiation Into iAstrocytes

Human skin biopsies were obtained from two healthy subjects after signing of informed consent approved by the Ethics Committee of Ospedale San Raffaele. Fibroblasts were isolated and reprogrammed to generate human iPSC clones with the Sendai virus technology (CytoTune-iPS Sendai Reprogramming Kit, Thermo Fisher Scientific) (19). iPSC clone characterization is described in (20). Human neural precursor cells (hiPSC-NPCs) were generated with the dual SMAD inhibition (SB431542/Dorsomorphin)/Hedgehog pathway activation (SAG/Purmorphamine)/WNT pathway activator (CHIR99021) and maintained in proliferation medium as described in (21). For astrocyte differentiation, the iNPCs were seeded at low density in Geltrex (Thermo Fisher Scientific)-coated T75 flasks (2 × 10^6^ cells/flask) for 24 h. The day after, proliferation medium was changed to DMEM supplemented with 1% antibiotics, 200 mM L-Glutamine, 100 mM Sodium Pyruvate (Thermo Fisher Scientific), 10% FCS and 0.3% N2 (22). Astrocytes were allowed to differentiate for several weeks, detached using trypsin and checked for morphology and marker expression at different time points. Phase contrast images for morphologic assessment were obtained at Leica DMIL LED microscope.

### Stimulation of Human iAstrocytes

Human iAstrocytes were incubated with 100 nM Fingolimod (FTY720-phosphate, Selleckchem), 100 nM Siponimod (BAF312, Selleckchem) or 1 μM NIBR-0213 (Merck) or vehicle (PBS or DMSO max 0.4% v/v) for 1 hour. Cells were then treated with IL1β (10 ng/ml, Thermo Fisher Scientific), IL17 (10 ng/ml, Peprotech) or S1P (100 nM, Echelon Biosciences). Incubation times were 1 h for S1P1 internalization assay, 30 min for NFκB assay, 1, 2 or 4 h for Nrf2 assay or 24 h for glutamate transporter assay. Cells were then processed for immunofluorescence and stained with appropriate primary antibodies. For the generation of astrocyte conditioned media iAstrocytes were pre-incubated with drugs, and then exposed to the inflammatory stimuli for 8 h. Astrocyte medium was replaced with fresh neuronal medium and, after additional 24 h culture, supernatants were collected, centrifuged to remove cell debris, and stored at −80°C. Before addition to primary neurons, astrocyte supernatants were diluted down to 1:4 with medium.

### RNA Extraction, cDNA Synthesis, and Qualitative PCR

Total RNA was extracted by Tri Reagent Solution (Thermo Fisher Scientific) and reverse transcribed using random hexamer primers and Superscript III reverse transcriptase (all from Thermo Fisher Scientific) following the manufacturers’ instructions. To remove contaminating DNA, RNA was treated with DNaseI enzyme (Thermo Fisher Scientific). As positive control, human peripheral blood mononuclear cells (PBMC) were isolated from a healthy donor as described in (10) and total RNA was extracted. Qualitative RT-PCR was performed using GoTaq G2 DNA polymerase (Promega) and dNTPs set (Thermo Fisher Scientific). The sequences of used primers are as follows: 5′-GGA GTA GTT CCC GAA GGA CC-3′ (sense) and 5′-TCT AGA ATC CAC GGG GTC TG-3′ (antisense) for S1P5 receptor (236-bp product), 5′-GAT GAC ATC AAG AAG GTG GTG AA-3′ (sense) and 5′-GTC TTA CTC CTT GGA GGC CAT GT-3′ (antisense) for glyceraldehyde-3-phosphate dehydrogenase (GAPDH) (246-bp product). Thirty-two cycles of amplification were performed at 94°C for 30 s, at 60°C for 30 s, and at 72°C for 1 min. PCR products were separated by electrophoresis on 2% agarose gel and visualized with SYBR Safe (Thermo Fisher Scientific) staining.

### Generation and Treatment of Primary Spinal Neurons

Primary spinal neurons were obtained from 16-day-old Sprague Dawley rat embryos as described (7, 23). Briefly, embryonal spinal cords, depleted of spinal root ganglia, were dissected, carefully minced and digested for 15 min at 37°C with 500 μg/ml DNAsi I (Roche) and 0.25% trypsin (Thermo Fisher Scientific) in L-15 medium (Thermo Fisher Scientific) supplemented with antibiotics. After digestion, tissue homogenate was washed 3 times with L-15 medium and finally cultured in Neurobasal medium (Thermo Fisher Scientific) supplemented with 10 ng/ml glial cell-derived neurotrophic factor (GDNF; Sigma), 20 ng/ml fibroblast growth factor (FGF; Peprotech), 50 μg/ml insulin (Sigma), B27 supplement (Thermo Fisher Scientific), 1% FCS (Euroclone), and 10 mM Glucose. Cells were seeded on poly-D-lysine and collagen (both from Sigma) coated glass coverslips. After 24 h 15 μM Cytosine b-D-arabinofurnoside (AraC; Sigma) was added to cultures and left for 4 days to eliminate contaminating microglia cells, astrocytes and oligodendrocytes. Neurons were exposed to astrocyte conditioned media for 8 h, then processed for immunofluorescence and stained with monoclonal antibody against β-tubulin. All nuclei were stained with DAPI. For assessment of neuronal counts, the numbers of DAPI positive nuclei were quantified and reported as percentage of control (neurons exposed to supernatants from vehicle-treated astrocytes; sCTRL). Neuronal network was measured by β-tubulin signal and expressed as percentage of controls.

## Immunofluorescence Experiments

Astrocytes or neurons were plated on coverslips, fixed in 4% PFA or MetOH, permeabilized with 0.2% Triton X-100 (Merck), blocked in PBS + 1% BSA (Merck) + 5% FCS and stained with primary antibodies. Then, cells were incubated with appropriate species-specific Alexa Fluor 488/594-conjugated secondary antibodies (Thermo Fisher Scientific), counterstained with 4′,6-diamidino-2-phenylindole (DAPI, Sigma) and mounted with fluorescent mounting medium (Agilent). The following primary antibodies were used: rabbit anti-GFAP (Agilent), mouse anti-nestin (Merck Millipore), mouse anti-vimentin (Abcam), rabbit anti-S100β (Abcam), rabbit anti-EDG1 (Santa Cruz Biotechnology), rabbit anti-EDG3 (Santa Cruz Biotechnology), mouse anti-IL1R (R&D), rabbit anti-IL17R (Santa Cruz biotechnology), rabbit anti- NFκB p65 (Abcam), rabbit anti-GLAST (Abcam), guinea pig anti-GLT1 (Merck Millipore), rabbit anti-Nrf2 (Abcam), mouse anti Neuronal Class III β-Tubulin (Covance). The following secondary antibodies were used: Alexa Fluor 488 donkey anti-rabbit IgG (H + L), Alexa Fluor 594 donkey anti-rabbit IgG (H + L), Alexa Fluor 488 donkey anti-mouse IgG (H + L), Alexa Fluor 594 donkey anti-mouse IgG (H + L), Alexa Fluor 488 goat anti-guinea pig IgG (H + L) (all from Thermo Fisher Scientific). Fluorescence images were captured at fluorescence microscope (Leica DM5500B) or Leica TCS SP5 confocal laser-scanning microscope equipped with 40× oil objective. LASAF and LASX softwares were used for image acquisition, and ImageJ (download at: http://rsbweb.nih.gov/ij/) software was used for image analysis. To quantify nuclear NFkB and Nrf2, DAPI images were converted to 8-bit, and regions of interest (ROIs) were generated to select (DAPI positive) nuclei. Then ROIs were applied to the corresponding NFkB or Nrf2 images, fluorescence thresholds were fixed on the unstimulated condition, and the fraction of positive nuclei was assessed (an example of analytical strategy is depicted for Nrf2 in Supplementary Figure S3). Similarly, the fraction of highly fluorescent astrocytes above the threshold was used to quantify cellular GLAST and GLT1 expression under the distinct conditions.

### Statistical Analyses

Data in figures are presented as mean ± standard deviation (SD) or standard error of the mean (SEM) as indicated in figure legends. The exact number of independent experiments performed is reported in figure legends. Unpaired *t*-test was performed to compare means. All *p*-values were two-sided and subjected to a significance level of 0.05. In figures, asterisks denote statistical significance as ^∗^*p* < 0.05; ^∗∗^*p* < 0.01; ^∗∗∗^*p* < 0.001. Statistical analyses were performed in Excel or GraphPad Prism.

## Results

### Generation and Characterization of Human iAstrocytes

**FIGURE 1:**
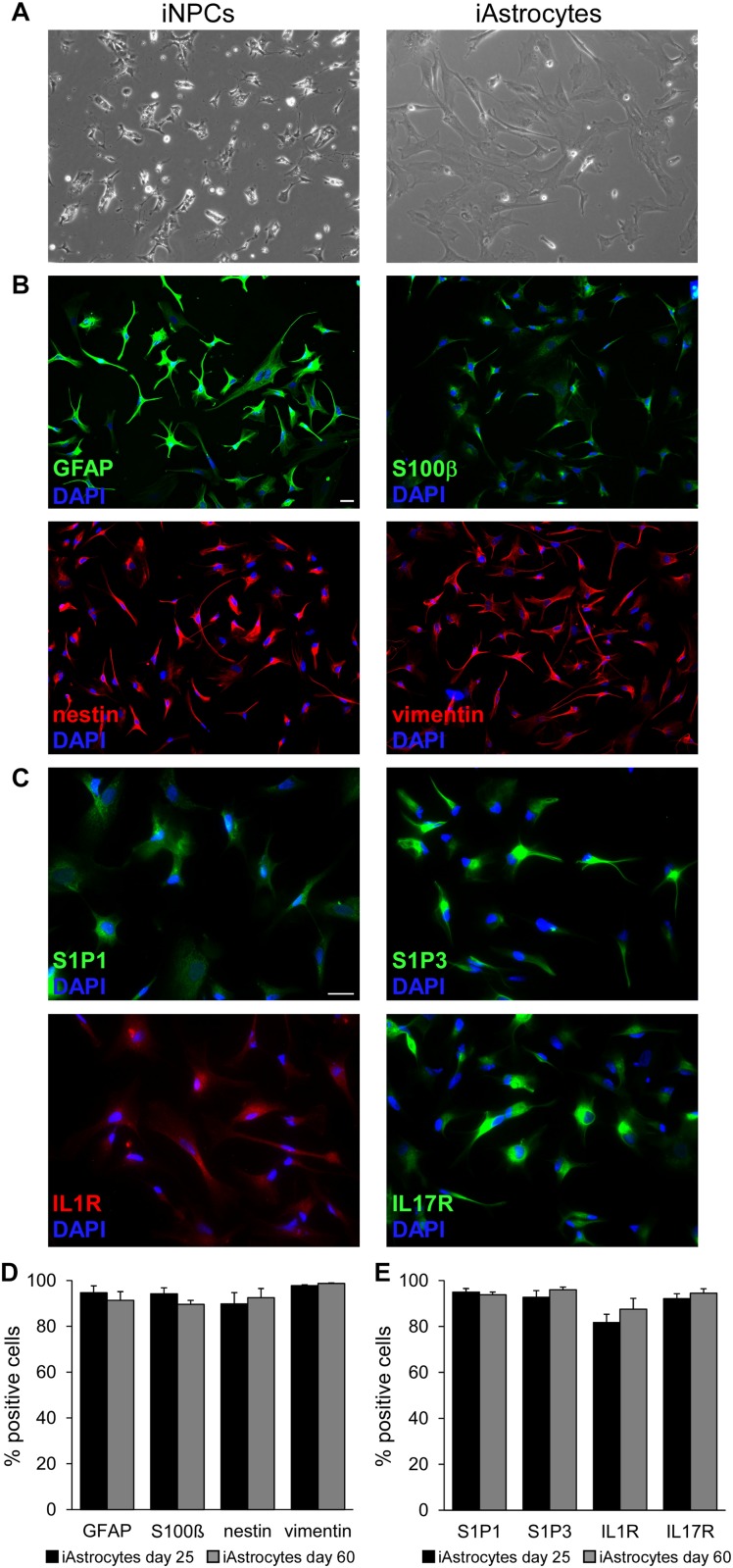
Characterization of iPSC-derived astrocytes. **(A)** Phase contrast images showing human iNPC (left panel) and human iAstrocytes (right panel). **(B,C)** Representative immunofluorescence stainings for GFAP, S100β, nestin, vimentin **(B)** and S1P1, S1P3, IL1R and IL17R **(C)** in human iAstrocytes. DAPI was used for nuclear staining. **(D,E)** Percentage of cells positive for astrocyte markers at two timepoints during differentiation. Reported quantifications were performed on three different human iAstrocytes preparations from the same iNPC cell line. Bars represent SEM. Same observations were recorded in human iAstrocytes from a second iNPC cell line. Scale bar = 30 μm.

Human fibroblasts were isolated from skin biopsies and reprogrammed to generate human iPSC clones, which were differentiated first in iPSC-derived NPCs and then into mature astrocytes (hereon called iAstrocytes). Cells were cultured for several weeks and sampled at different time points to assess their morphology and phenotype. First, we observed a significant change in cell size and morphology during the differentiation process from iNPC, as human iAstrocytes clearly showed an increment in cell size and acquired the typical morphology of astroglia (Figure 1A). Immunofluorescence experiments confirmed that classical astrocyte markers were highly expressed in all analyzed cultures. In fact, iAstrocytes were positive for GFAP, S100ß, nestin and vimentin that remained expressed at high levels also at advanced culture stages (Figures 1B,D). As reactive astrocytes in MS lesions display coordinated upregulation of receptors for inflammatory cytokines (IL1R, IL17R) and the lipid mediator S1P (S1P1 and S1P3) (7), we checked protein expression of these receptors on human iAstrocytes and observed strong positivity for all of them (Figures 1C,E), indicating that our *in vitro* human cell model mimics the phenotype of the reactive glia cell in the human pathological tissue and may be responsive to inflammatory mediators. Human iAstrocytes did not express transcripts for S1P5, another possible target of BAF312 or FTY720 (Supplementary Figure S1).

### BAF312 Blocks Inflammatory Activation of iAstrocytes and Supports Maintenance of Glutamate Transporters

We set up *in vitro* assays with our human cell system to study the effects of BAF312 and FTY720 on different astrocyte functions.

As these drugs were shown to induce rapid S1P1 internalization in rodent astrocytes (24, 25), we checked this phenomenon in our cells and confirmed that, differently from control cells, iAstrocytes displayed intracellular S1P1 aggregates with perinuclear distribution when exposed to FTY720 or BAF312 for 1 h (Supplementary Figure S2).

**FIGURE 2:**
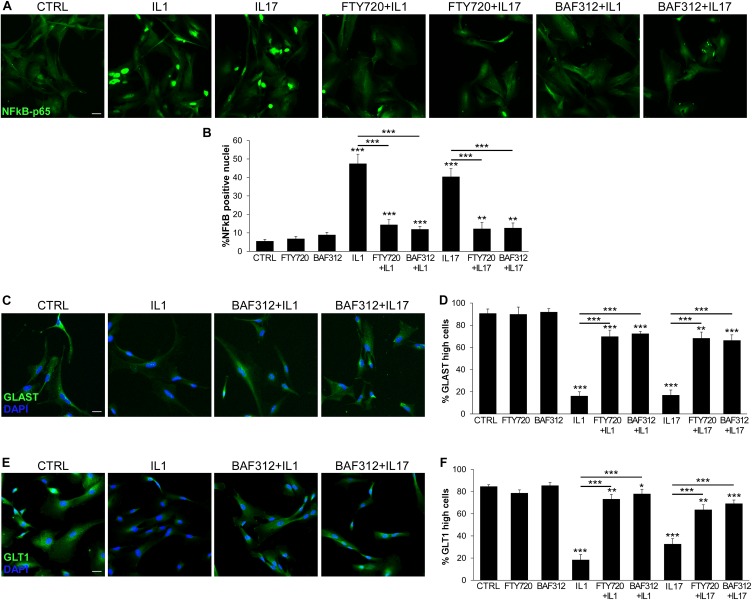
BAF312 inhibits NFκB nuclear translocation and maintains glutamate transporters expression in astrocytes exposed to inflammatory cytokines. **(A)** Representative immunofluorescence stainings for NFκB-p65 in human iAstrocytes stimulated with cytokines alone or after pre-incubation with BAF312. **(B)** Graph reports the percentage of NFκB-p65 positive nuclei in human iAstrocytes under distinct conditions. **(C–F)** Representative immunofluorescence stainings for GLAST **(C)** and GLT1 **(E)** in human iAstrocytes and relative quantifications **(D,F)** under distinct conditions. Representative images are shown. DAPI was used for nuclear staining. Data are shown as mean ± SD of a representative experiment out of three independent experiments. Scale bars: 30 μm. *p < 0.05, **p < 0.01, ***p < 0.001.

NFκB is a key transcription factor in cytokine and S1P signaling, and plays a pivotal role in the amplification of inflammatory and neurodegenerative processes (5, 26). We verified whether our human iAstrocytes activated NFκB in response to inflammatory cues and studied the effect of S1P signaling modulators on astrocyte behavior. As shown in Figures 2A,B, IL1 or IL17 strongly induced nuclear translocation of NFκB-p65 in human iAstrocytes, however, this effect was blocked by astrocyte exposure to BAF312 or FTY720.

Maintenance of extracellular glutamate concentrations below neurotoxic levels is a critical function of glial glutamate transporters GLAST and GLT1 (27). Both transporters were expressed on resting iAstrocytes (CTRL; Figures 2C,E), but strongly downregulated in cells exposed to inflammatory cytokines for 24 h (Figures 2C–F). Differently, cells treated with BAF312 or FTY720 maintained high GLAST and GLT1 expression even under inflammatory conditions (Figures 2C–F).

**FIGURE 3:**
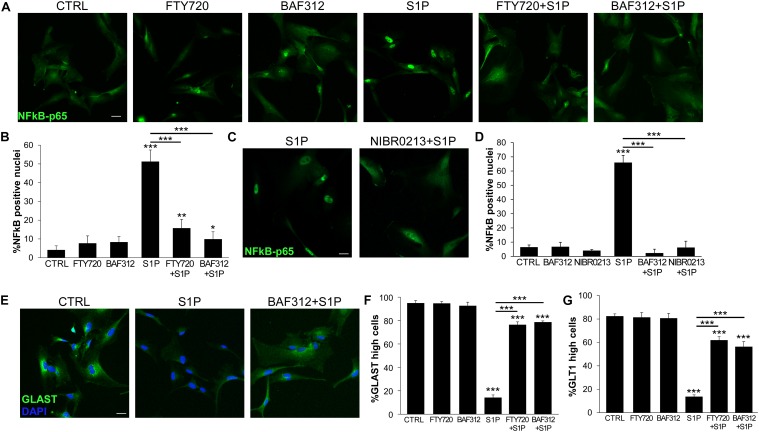
S1P1 targeting is sufficient to support physiological astrocyte functions. **(A)** Representative immunofluorescence stainings for NFκB-p65 in human iAstrocytes stimulated with S1P alone or after pre-incubation with FTY720 or BAF312 and relative quantifications **(B)**. **(C)** Fluorescence images for NFκB-p65 in human iAstrocyte cultures stimulated with S1P eventually in the presence of NIBR-0213. Percentage of NFκB-p65 positive nuclei is reported in **(D)**. **(E)** Representative immunofluorescence stainings for GLAST in human iAstrocytes exposed to vehicle (CTRL), S1P alone or after pre-incubation with BAF312. **(F,G)** Frequency of GLAST **(F)** or GLT1 **(G)** high positive cells under the different experimental conditions. DAPI was used for nuclear staining. Data are shown as mean ± SD of a representative experiment out of three independent experiments. Scale bars: 30 μm. *p < 0.05, **p < 0.01, ***p < 0.001.

Astrocytes may react to the mediator S1P via S1P1 and S1P3. While FTY720 targets both receptors, BAF312 is selective for S1P1 only, leaving open the possibility of responding to S1P via S1P3. To check this hypothesis we used the NFκB assay to measure astrocyte activation in response to S1P and differentially interfered with S1P signaling via FTY720 or BAF312. As shown in Figure 3, selective blockade of S1P1 and not S1P3 with BAF312 achieved similar, strong inhibition of S1P-induced NFκB-p65 translocation to that exerted by FTY720 (Figures 3A,B). As additional control, iAstrocytes were treated with NIBR-0213, a potent and selective S1P1 antagonist (28), and S1P-mediated NFκB-p65 nuclear translocation was assessed. Similarly to BAF312, specific inhibition of S1P1 via NIBR-0213 abolished S1P-evoked NFκB-p65 translocation (Figures 3C,D). S1P also downregulated protein expression of glutamate transporters on iAstrocytes (Figures 3E–G), however, this process was equally hindered by FTY720 and BAF312 (Figures 3E–G).

All together these data indicate that triggering of inflammatory signaling cascades in glia cells may be prevented by S1P receptor modulators, and that S1P1 targeting via BAF312 is sufficient to directly dampen inflammatory activation via other S1P receptors and support physiological astrocyte functions.

### BAF312 Induces Nrf2 Activation in Human iAstrocytes

**FIGURE 4:**
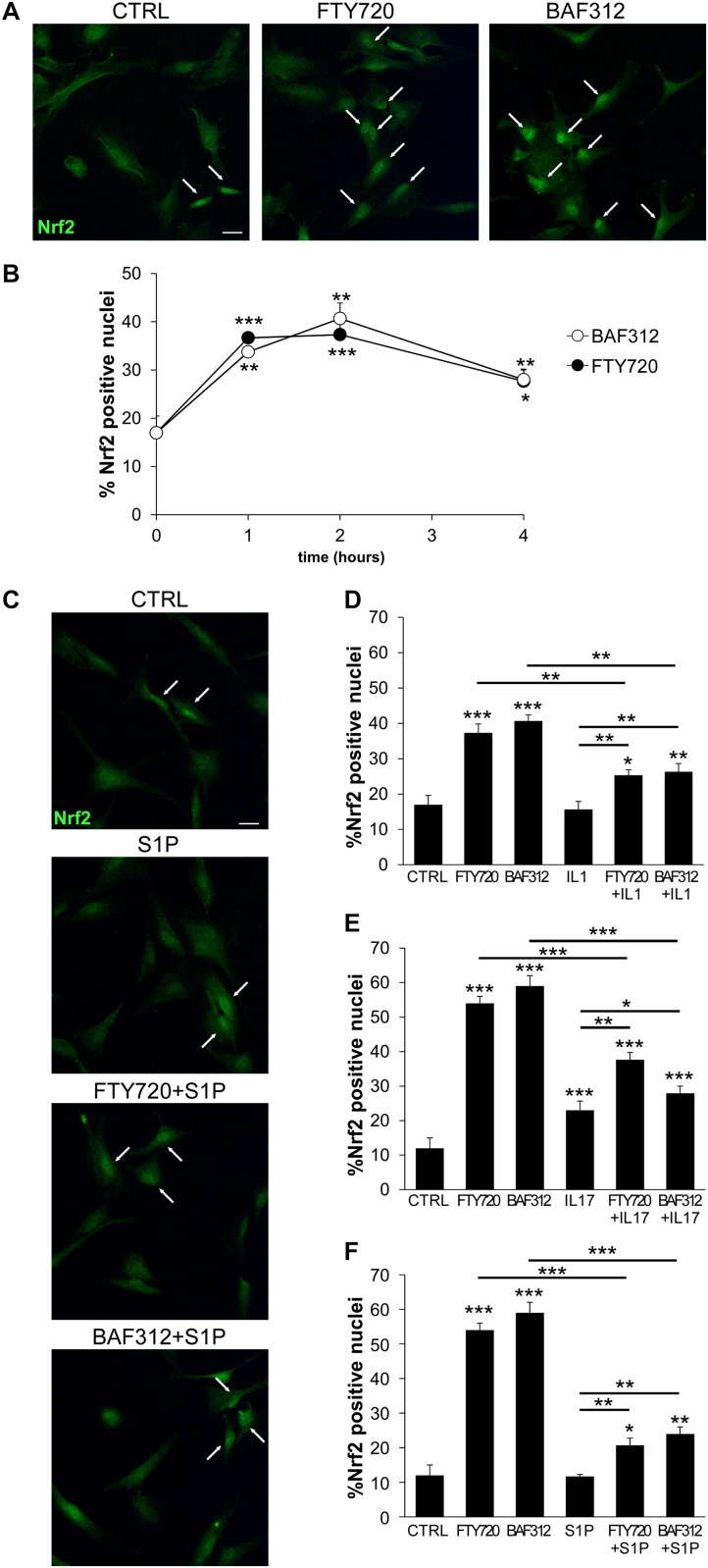
S1P receptor modulators induces Nrf2 activation in glial cells. **(A)** Representative immunofluorescence stainings for Nrf2 in human iAstrocytes exposed for 1 hour to FTY720 or BAF312. **(B)** Frequency of Nrf2 positive nuclei in human iAstrocytes stimulated with drugs at different time points. **(C)** Representative Nrf2 immunofluorescence stainings in iAstrocytes exposed for 1 hour to S1P alone or after 1 hour pretreatment with drugs. **(D–F)** Frequency of Nrf2 positive nuclei in human iAstrocytes treated with drugs alone or in the presence of IL1 **(D)**, IL17 **(E)** or S1P **(F)**. Data are shown as mean ± SD of a representative experiment out of 2–3 independent experiments. In **(A)** and **(C)** white arrows highlight Nrf2 positive nuclei. Scale bar: 30 μm. *p < 0.05, **p < 0.01, ***p < 0.001.

Activation of the transcription factor Nrf2 represents a key checkpoint for cellular antioxidant responses and its induction in astrocytes may confer neuroprotection during neuroinflammation (29). To ascertain whether BAF312 and FTY720 may regulate Nrf2 activation in glial cells, human iAstrocytes were stimulated with the drugs over a few hours and assessed for Nrf2 expression by immunofluorescence. Under resting conditions, iAstrocytes displayed mainly cytoplasmic Nrf2 expression (Figure 4A), however, exposure to S1P modulators for 1 h significantly increased Nrf2 nuclear levels (Figures 4A,B), an effect that persisted over time (Figure 4B). We then checked whether this action was maintained under inflammatory conditions, and noted that Nrf2 induction was significantly reduced in the presence of IL1, IL17 or S1P (Figures 4C–F). These experiments indicate that S1P receptor modulators may directly activate protective responses in glial cells via Nrf2, but that their efficacy may be affected upon neuroinflammation.

### BAF312 Hampers Astrocyte-Induced Neurodegeneration

**FIGURE 5:**
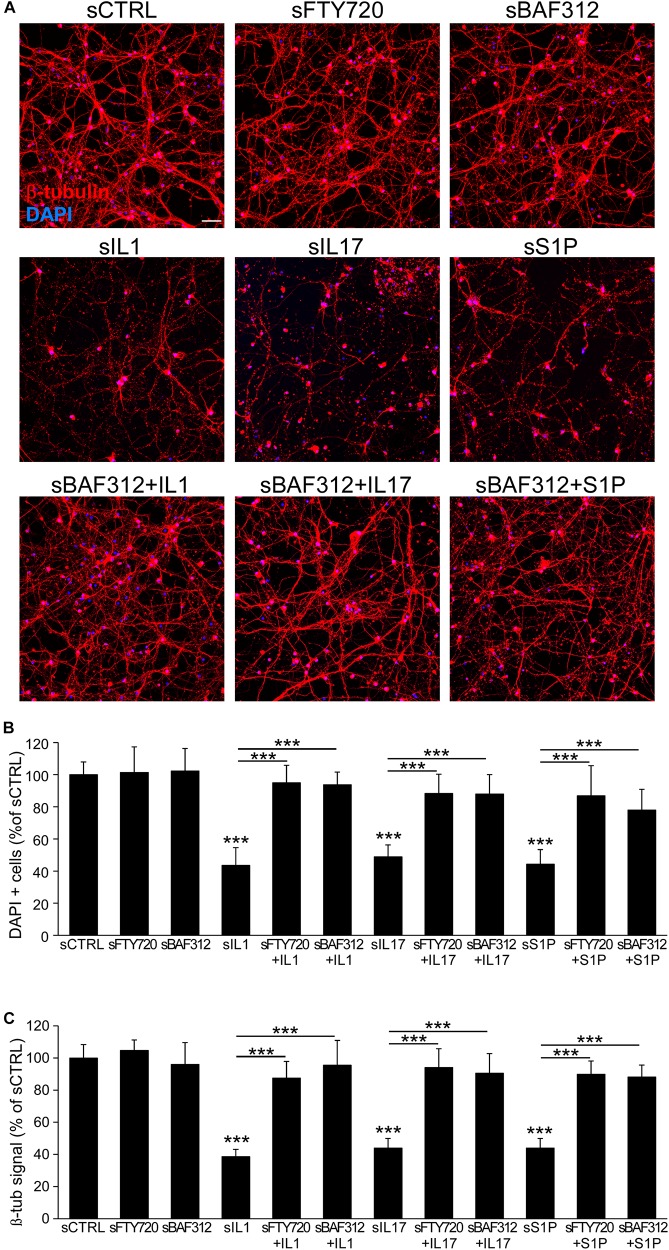
BAF312 blocks neurodegeneration induced by astrocyte responses to cytokines and S1P. **(A)** Representative immunofluorescence stainings for β-tubulin and DAPI of spinal neurons after exposure to media generated from vehicle (sCTRL)-, FTY720- or BAF312- treated astrocytes (upper panels), IL1-, IL17- or S1P-activated astrocytes (middle panels) or eventually from astrocytes treated with BAF312 (lower panels). **(B,C)** Quantification of cell number **(B)** and β -tubulin signal **(C)** expressed as percentage respect to supernatants from control-treated cultures (sCTRL). Graphs show cumulative results from three independent experiments. Data are represented as mean ± SEM. Scale bars: 50 μm. ***p < 0.001.

To test the overall impact of astrocyte mediators on neurons, we exposed human iAstrocytes to the drugs and then to the inflammatory mediators for 8 h, changed the medium to remove stimuli and collected the supernatants after a further 24 h culture. Astrocyte-conditioned media were then added to pure cultures of spinal neurons, which were then assessed for cell number and morphology via DAPI and β-tubulin stainings. While supernatants from non-treated (sCTRL) or FTY720- or BAF312- treated cultures (sFTY720, sBAF312) did not affect neuronal survival and network integrity, conditioned media from human iAstrocytes stimulated with the inflammatory factors (sIL1, sIL17, and sS1P) triggered robust degenerative responses characterized by neuronal loss and neurite fragmentation (Figures 5A–C). However, when astrocyte media were generated in the presence of BAF312 or FTY720, their addition to spinal neurons did not trigger neurodegeneration despite astrocyte exposure to the inflammatory mediators (Figures 5A–C), indicating that astrocyte targeting by S1P receptor modulators may rescue neurons from astrocyte-induced degeneration.

## Discussion

In this study, we provided *in vitro* evidence of neuroprotective effects of BAF312 via the human astrocyte during inflammation. In particular, we (i) generated human fibroblast-derived astrocytes to measure pharmacological effects of S1P receptor modulators; (ii) addressed the direct impact of BAF312 on NFkB activation and glutamate transporters in astrocytes; (iii) demonstrated the direct activation of Nrf2 by S1P receptor modulators; and (iv) performed side-by-side comparisons between BAF312 and FTY720 in all *in vitro* assays. Importantly, BAF312 action on the astrocyte was strong enough to inhibit neurodegeneration triggered by glial mediators generated during neuroinflammation.

The recent results about the efficacy of the S1P receptor modulator BAF312 in the treatment of secondary progressive MS indicate that the slowdown of disability progression is associated with significant effects on lesion enlargement and brain atrophy (18), thus on the neuropathological, degenerative components of SP-MS due to oligodendrocyte injury and axonal degeneration aggravated by dysregulated activity of CNS resident cells (30). Astrocytes may contribute to these processes by several mechanisms, including the release of inflammatory cytokines and cytotoxic factors and the formation of a dense glial scar inhibiting tissue repair (31, 32). *In vivo* findings from experimental neuroinflammation support the hypothesis of protective effects of BAF312 in the CNS (33). In fact, direct administration of the drug into the CNS of animals with experimental MS ameliorates disease expression, and reduces CNS inflammation and loss of GABAergic signaling (33). This action is accompanied by shrinkage of astrogliosis *in vivo*, suggesting an impact on the inflammatory activation of the astrocyte (33). Astrocytes upregulate S1P receptors S1P1 and S1P3 *in vitro* and *in vivo* under inflammatory conditions (7, 34), thus they may become target of S1P signaling modulators. BAF312 may directly induce *in vitro* glial Ca^2+^ levels and ERK phosphorylation mainly via S1P1 receptor (25). Our *in vitro* data corroborate the evidence for BAF312 action on several distinct astrocyte functions, and identify Nrf2 and NFκB as crucial transcription factors regulated by BAF312 in astrocytes. Whilst glial Nrf2 induces *in vivo* anti-oxidant, anti-inflammatory and neuroprotective responses (35), astrocytic NFκB is primarily involved in pro-inflammatory reactions, scar formation and neurodegeneration (36–39). Thus, the balance between these two pathways is critical for regulation of cellular responses to stress and inflammation. It is relevant to underline that functional S1P signaling is necessary for cytokine-evoked astrocyte activation, as its targeting by FTY720 completely impairs glial NFκB translocation in response to IL1 and IL17 (7). Here we show for the first time that the S1P modulator BAF312 rapidly induces Nrf2 nuclear translocation in glia cells, and that this phenomenon is paralleled by the blockade of NFκB activation under inflammatory conditions. Considering that S1P concentration is high in blood (>100 nM) (40) and that levels around 100 nM may be plausibly reached in the inflamed CNS due to blood-brain barrier breakdown, to interfere with S1P signaling we employed 100 nM BAF312 or active FTY720 in our *in vitro* tests. No information is available about the concentration reached by the two drugs in the human CNS, while it is known that BAF312 and active FTY720 concentrations are, respectively, around 60 and 5 nM in plasma of human treated subjects (41, 42). Notably, evidences from mouse models indicate that drug levels in the CNS exceed those in blood severalfold, and that FTY720 accumulates even more in the inflamed vs. healthy CNS (43, 44). Here, we report that BAF312 and FTY720 display comparable efficacy in parallel *in vitro* tests, confirming the crucial pathogenic role of S1P signaling in astrocyte function and implying that the drugs may result equally potent on glial cells, assuming that the levels of active FTY720 are similar to those reached by BAF312.

Although S1P signaling in astrocytes can be triggered by two (S1P1 and S1P3) receptors, S1P1 appears to play a major role in glial functions. In fact, S1P1-selective agonism reduces astrogliosis in experimental MS with similar efficacy to FTY720, which targets several S1P receptors (45). Further, transgenic mice with selective removal of S1P1 from GFAP-expressing cells develop milder neuroinflammatory disease and do not respond to FTY720 treatment (45). Here we show that, in our *in vitro* human system, S1P signaling via S1P1 is necessary and sufficient to modulate astrocyte behavior. In fact, glial responses to S1P are efficiently and equally inhibited by BAF312 (which targets S1P1) and FTY720 (which targets both S1P1 and S1P3). Moreover, treatment with the potent and selective S1P1 antagonist NIBR-0213 is sufficient to abolish NFκB activation.

Internalization of S1P1 receptor following ligand binding has been observed in cells artificially overexpressing S1P1 (45, 46). This phenomenon has been visualized in primary rodent astrocytes exposed to nanomolar doses of FTY720 or to micromolar BAF312 (24, 25), while no evidence was available for human astrocytes. Here we show that FTY720 and BAF312 induce formation of intracellular S1P1 aggregates with perinuclear distribution in our cells, thus demonstrating internalization of physiological levels of S1P1 in human glia. Notably, internalized S1P1 receptors may maintain signaling activity following exposure to FTY720 and not S1P, suggesting persistent agonism mediated by the drug (24, 46). On the other hand, our studies indicate that S1P receptor modulators are functional antagonists of S1P and cytokine signaling. This action may thus result either from the loss of surface S1P1 receptor or from the interference of internalized S1P1 activity with inflammatory signaling, an issue which deserves further investigation.

A pathological consequence of neuroinflammation is the dysfunction of glutamatergic transmission due to malfunctioning of glutamate transport. Glutamate is the main excitatory neurotransmitter in the CNS, but excessive glutamate accumulation in the synaptic and extra-synaptic spaces leads to neuronal death through a process called excitotoxicity (47). Under physiological conditions glutamate clearance from the extracellular milieu is primarily achieved by astrocytes via the glutamate transporters GLAST and GLT1 (48), whose levels, however, become low under inflammatory state (49, 50). The beneficial effect of FTY720 in experimental MS has been associated with restoration of glial glutamate transporters (51). Our study proves that inflammatory cytokines and S1P indeed downregulate GLAST and GLT1 proteins in astrocytes, and that BAF312 or FTY720 directly support the maintenance of these transporters. This implies that S1P signaling modulators may restore proper glutamate buffering by astrocytes back to physiological levels.

Astrocyte activation is crucial in driving inflammation-induced neurodegeneration. In fact, S1P- or cytokine-activated astrocytes release factors that trigger neuronal death, as nitric oxide (7) or reactive oxygen species (52). Importantly, blockade of S1P signaling in glia cells by FTY720 hampers NO release in response to S1P and inflammatory cytokines, and prevents from astrocyte-induced neuronal death (7). Similarly to what shown for FTY720 (7), our experiments on primary cultures of spinal neurons demonstrate that neurotoxicity mediated by conditioned media from activated astrocytes is abolished when astrocytes are exposed to BAF312. This final evidence unequivocally confirms that the net result of the modulation of S1P signaling in the astrocyte is indeed the blockade of astrocyte-mediated neurodegeneration.

## Conclusion

In conclusion, our investigation highlights indirect neuroprotective properties for BAF312 via targeting S1P-S1P1 axis in glia cells.

## Data Availability Statement

All datasets generated for this study are included in the article/Supplementary Material.

## Ethics Statement

The participants to the study provided their written informed consent approved by the Ethics Committee of Ospedale San Raffaele, Milan.

## Author Contributions

EC and CB performed *in vitro* experiments and statistical analyses. AD performed the iAstrocyte differentiation from human iNPCs. FR and LO generated and provided the human iNPCs. EC and CF analyzed the data and wrote the manuscript. GC and GM discussed the project and results. GM provided reagents. CF conceived and designed the experiments. All authors read and approved the submitted version.

## Conflict of Interest

GC received consulting and speaking fees from Novartis, Teva Pharmaceutical Industries Ltd., Teva Italia Srl, Sanofi Genzyme, Genzyme Corporation Genzyme Europe, Merck KGgA, Merck Serono SpA, Celgene Group, Biogen Idec, Biogen Italia Srl, F. Hoffman-La Roche, Roche SpA, Almirall SpA, Forward Pharma, Medday, and Excemed. CF received grant support from Novartis and FISM related to the work. The remaining authors declare that the research was conducted in the absence of any commercial or financial relationships that could be construed as a potential conflict of interest.

## Footnotes

1 https://www.ema.europa.eu/en/medicines/human/summaries-opinion/mayzent
