# Supplementary figures and images for: Siponimod (BAF312) Activates Nrf2 While Hampering NFκB in Human Astrocytes, and Protects From Astrocyte-Induced Neurodegeneration
Source: Front Immunol. 2020 Apr 8;11:635. doi: 10.3389/fimmu.2020.00635 (PMC7156595; doi:10.3389/fimmu.2020.00635)

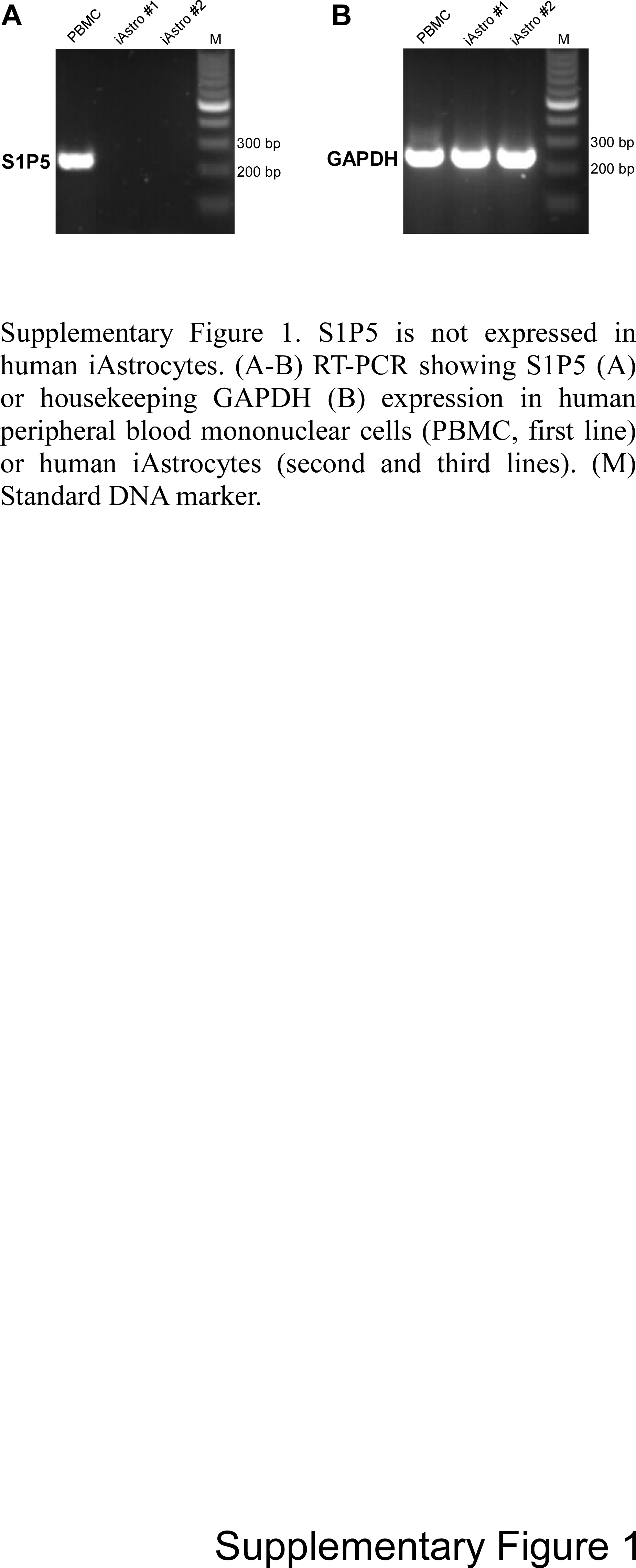

Supplement: Supplementary file 1 [file Image_1.TIF]

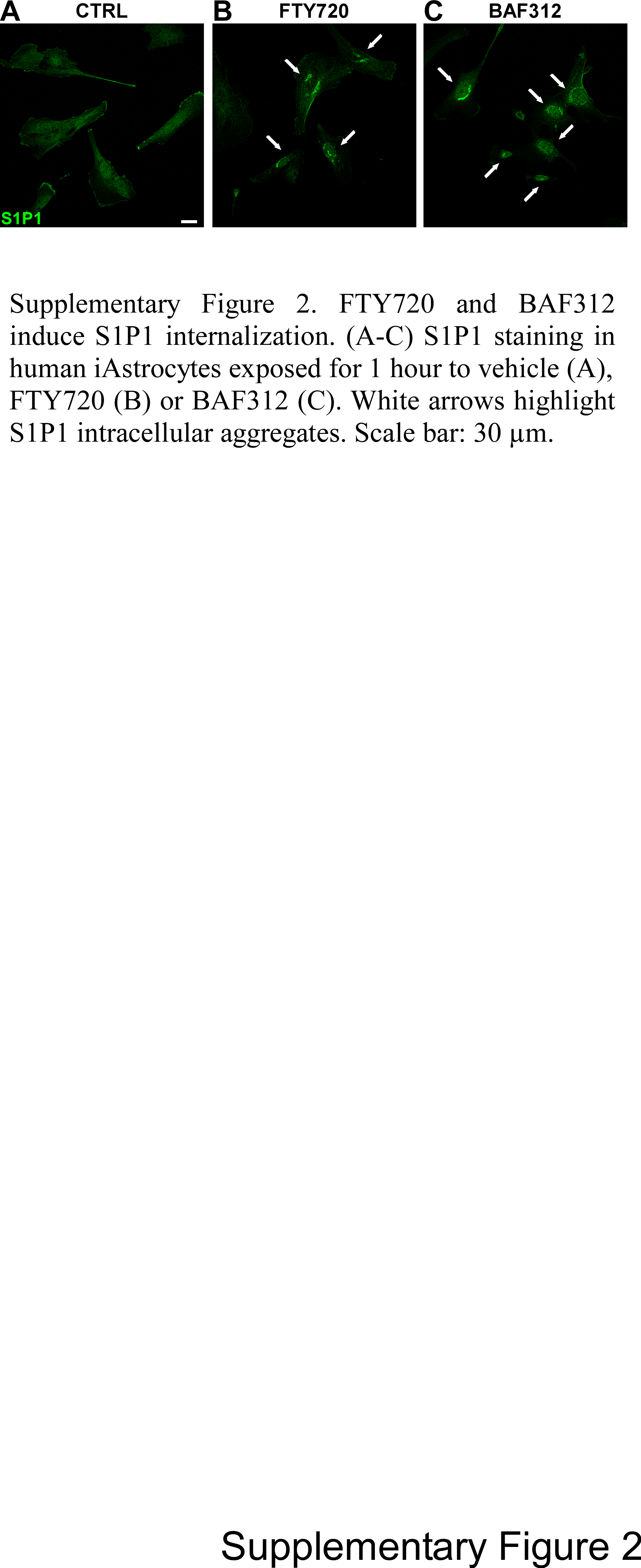

Supplement: Supplementary file 2 [file Image_2.TIF]

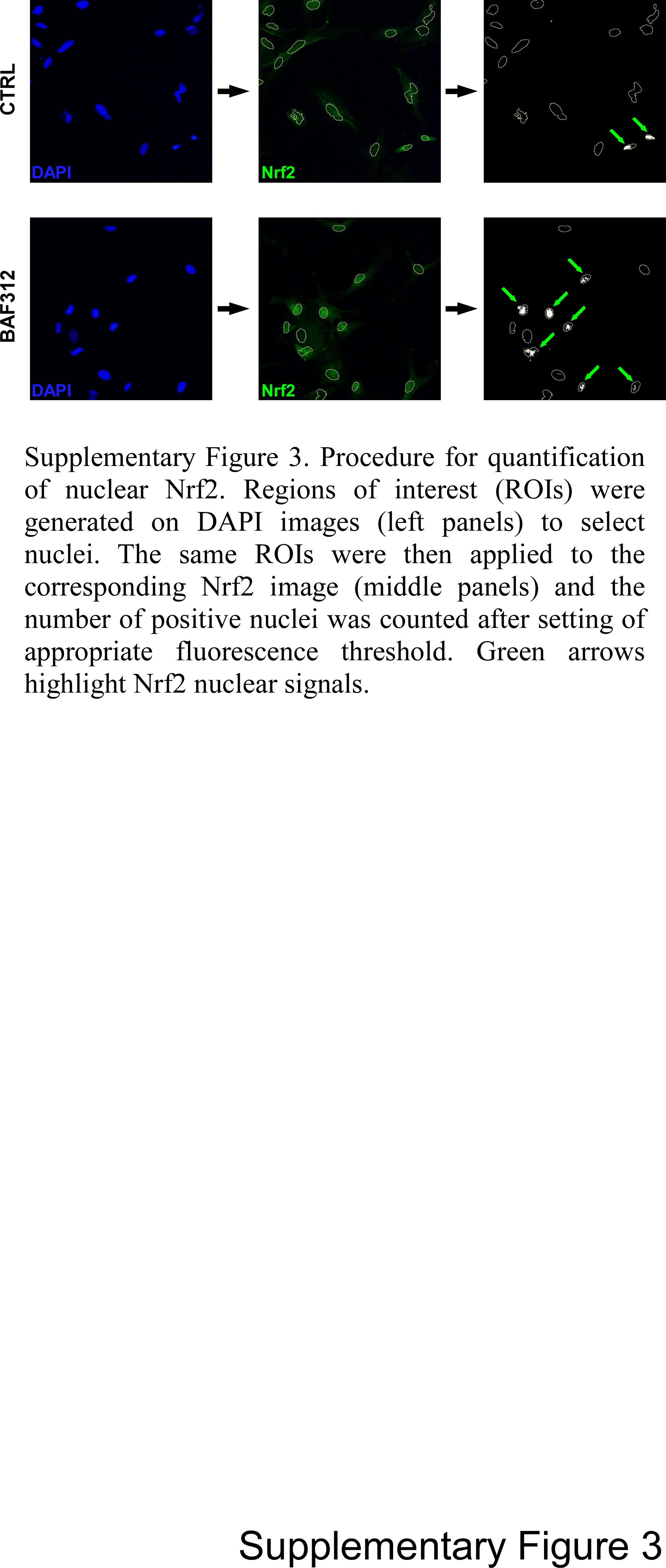

Supplement: Supplementary file 3 [file Image_3.TIF]
